# Aminopeptidase A Effect on Angiotensin Peptides and Their Blood Pressure Action

**DOI:** 10.3390/ijms26146990

**Published:** 2025-07-21

**Authors:** Peter Forster, Jan Wysocki, Yasemin Abedini, Tilman Müller, Minghao Ye, Carlos M. Ferrario, Daniel Batlle

**Affiliations:** 1 Division of Nephrology and Hypertension, Department of Medicine, Northwestern University Feinberg School of Medicine, Chicago, IL 60611, USA; peter.forster@charite.de (P.F.); j-wysocki@northwestern.edu (J.W.); yasemin.abedini-nadjafabadi@charite.de (Y.A.); tilmanmue@gmail.com (T.M.); sunshine.yao99@gmail.com (M.Y.); 2 Charité-Universitätsmedizin Berlin, 10117 Berlin, Germany; 3 Department of Surgery, Wake Forest University School of Medicine, Winston-Salem, NC 27101, USA; cferrario45@gmail.com

**Keywords:** angiotensin, renin angiotensin system, angiotensin II, angiotensin-(1-12), aminopeptidase A

## Abstract

Aminopeptidase A (APA) cleaves a single aspartate residue from the amino terminus of peptides within the renin angiotensin system (RAS). Since several RAS peptides contain an N-terminal aspartate, we developed an assay to evaluate the effect of recombinant APA on the cleavage of Ang I, Ang II, Ang-(1-7), Ang-(1-9), and Ang-(1-12). The latter peptide has been proposed to be a functional Ang II-forming substrate with a hypertensive action attributable to the formed Ang II acting on AT1 receptors. Here we investigated the following: (a) the hydrolytic action of APA on Ang-(1-12), Ang I (1-10), Ang-(1-9), Ang II and Ang-(1-7) and (b) whether Ang-(1-12) pressor activity is altered by recombinant APA (r-APA) or genetic APA deficiency. We found that (a) r-APA cleaves the N-terminal aspartate of not only Ang II but also [Ang-(1-12), Ang I (1-10), Ang-(1-9)] and [Ang-(1-7)]; (b) the pressor activity of Ang-(1-12) was abolished in the presence of Lisinopril or Telmisartan; (c) r-APA significantly attenuated the pressor activities of infused Ang I and Ang II but not Ang-(1-12); and (d) r-ACE2 also did not attenuate the pressor effect of infused Ang-(1-12). Thus, in addition to increasing blood pressure indirectly via the formation of Ang II, Ang-(1-12) increases blood pressure by an Ang II-independent mechanism. We conclude that APA has an antihypertensive effect attributable to rapid degradation of Ang II, and this action may have a therapeutic potential in forms of hypertension that are Ang II-dependent. In addition, APA metabolizes Ang-(1-12), a peptide that has a prohypertensive action, in part, as a source of Ang II formation but also by a yet to be determined action independent of Ang II.

## 1. Introduction

The prevalence of hypertension has remained a formidable challenge to public health systems worldwide over the past decades [1,2,3]. One of the pivotal systems implicated in the regulation of blood pressure is the renin angiotensin system (RAS), with its intricate interplay of hormones (e.g., Angiotensin [Ang] I and Ang II), enzymes (e.g., renin, angiotensin-converting-enzyme [ACE], and aminopeptidase A [APA]), and receptors (e.g., angiotensin type 1 [ATR1] and type 2 [ATR2] receptors) making it a primary focus of therapeutic development [4,5]. Ang II is a potent vasoconstrictor considered the main effector peptide of the RAS. However, several other angiotensin peptides beyond Ang II might fine-tune the impact on blood pressure. In addition, the activity of RAS enzymes that either form or catabolize Ang II and target other vasoactive angiotensin peptides may also play an essential role in blood pressure regulation. Preclinical studies have generated a new interest in developing therapeutic approaches targeting Ang II degradation [6,7,8,9,10]. One of those enzymes is aminopeptidase A (APA) which metabolizes Ang II by cleaving a single aspartate residue from the N-terminus [11,12,13,14]. This enzyme also degrades Ang I by the same mechanism [11,12,13,14]. Genetic deficiency of APA in mice is associated with ultrastructural glomerular pathology [11] and increased blood pressure [11,15]. Furthermore, hypertensive animal models have documented significant antihypertensive responses following exposure to the centrally acting APA inhibitors firibastat and NI956 [16].

In filling a void regarding the potential of using angiotensin peptides with a truncated N-terminus for therapeutic approaches, we evaluated whether hydrolytic cleavage of Asp by recombinant mouse APA (r-APA) in mice with and without a genetic APA deficiency [17] modified the blood pressure response to infusion of Ang-(1-12) and the downstream angiotensins—Ang I, Ang-(1-9), Ang II, and Ang-(1-7).

## 2. Results

### 2.1. In Vitro Assessment of Cleavage of N-Terminal Aspartate from Various Angiotensin Peptides by Recombinant APA

**Figure 1:**
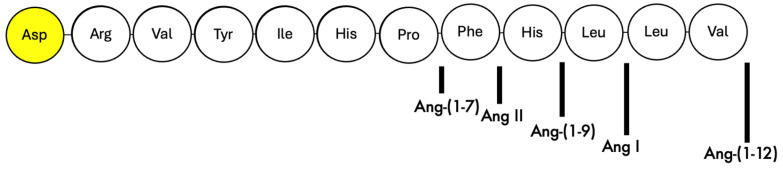
Amino acid sequence of several Angiotensin peptides with an N-terminal aspartate residue (yellow-highlighted circle).

An in vitro assay was used to evaluate the hydrolytic activity of r-APA on the included angiotensins (Figure 1).

**Figure 2:**
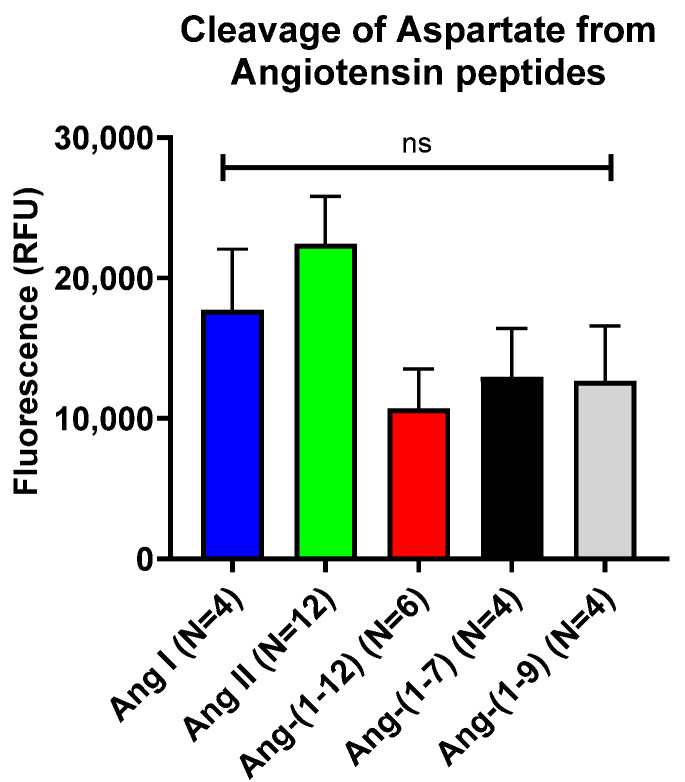
Cleavage of N-terminal aspartate from various angiotensin peptides by murine recombinant aminopeptidase A (rAPA) measured by fluorescence formation using the in vitro assay described in the Methods. Each bar represents the mean ± standard error of 4–12 experiments of each of the peptides tested; ns—not significant.

In the in vitro assay, the murine rAPA (mrAPA) showed a substantial cleavage of aspartate from Ang I (17,735 ± 4316 RFU, N= 4), Ang II (22,437 ± 3358 RFU, N = 12), Ang-(1-12) (10,719 ± 2825 RFU, N = 6), Ang-(1-7) (12,964 ± 3461 RFU, N = 4), and Ang-(1-9) (12,662 ± 3930 RFU, N = 4) as evidenced by Aspartate-driven fluorescence formation (Figure 2 and Supplemental Figure S1). By ANOVA, there was no significant difference in fluorescence formation between those five Ang peptides. As expected, negligible free-aspartate formation was detected for Ang A (48 ± 46 RFU, N = 6) and Alamandine (−549 ± 194 RFU, N = 3) (Supplemental Figure S1) which both lack aspartate at the N-terminus [18,19]. Like mrAPA, human rAPA cleaved aspartate from Ang II, Ang I, Ang-(1-7), Ang-(1-9), and Ang-(1-12) with comparable efficiencies; and also similar to mrAPA, there was a lack of fluorescence formation when human rAPA was incubated in the presence of either Ang A or Alamandine (Supplemental Figure S1). Expectedly, incubation with rACE2, which is a monocarboxypeptidase, did not cleave the N-terminal aspartate as evidenced by no appreciable fluorescence formation from any of the angiotensins tested (Supplemental Figure S1). In addition, the APA inhibitor, amastatin (10^−5^ M), markedly reduced fluorescence formation when mrAPA was incubated with Ang II (Supplemental Figure S2). This finding confirms the specificity of the assay.

### 2.2. Effect of APA-Cleavable Ang Peptides on Systolic Blood Pressure

**Figure 3:**
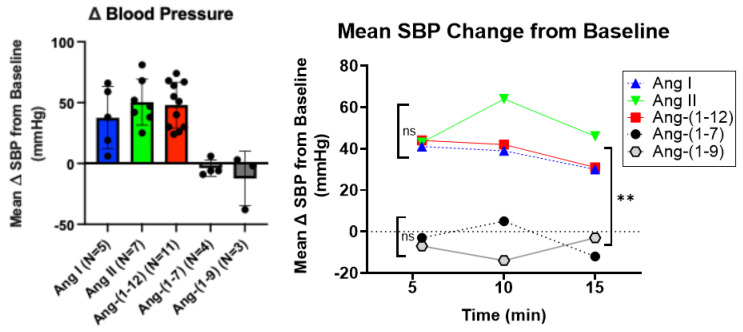
Systolic blood pressure (SBP) and mean SBP change from baseline after i.p. injection of different Ang peptides (0.2 μg/g body weight). Changes in systolic blood pressure (SBP) from the baseline in mice after a single i.p. injection of five Ang peptides (Ang I, Ang II, Ang-(1-12), Ang-(1-7), and Ang-(1-9) each at 0.2 μg/g body weight). (**Left**): Average difference (Δ) between the SBP measured during 5 min before and the SBP recorded during 5 min after an intraperitoneal (IP) administration of the angiotensin peptides (N = 3 to N = 11). (**Right**): Average differences (Δ) in SBP between baseline (SBP measured during 5 min before i.p. injection of the Ang peptide) and SBP measured 5 min, 10 min, and 15 min after i.p. injection of the Ang peptide; analyzed by 2-way ANOVA and Tukey post-hoc comparisons; ns—not significant differences between Ang-(1-7) and Ang-(1-9) and between Ang I, Ang II, and Ang-(1-12); **—denotes *p* < 0.01 or *p* < 0.001 regarding Ang-(1-7) and Ang-(1-9) vs. Ang I, Ang II, and Ang-(1-12) (N = 3 to N = 5).

Out of the five Ang peptides, proven to be rAPA substrates in the in vitro assay, (Figure 2), only Ang-(1-12), Ang I, and Ang II exhibited a marked increase in systolic blood pressure (SBP) when administered i.p. to male C57BL/6J mice (Figure 3). Comparative bolus injections of Ang-(1-9) and Ang-(1-7) had no effect on blood pressure even though both angiotensins are degraded by rAPA.

### 2.3. Murine Recombinant Aminopeptidase a Reduces Acute Hypertension Induced by Ang I, Ang II, and Ang-(1-12)

As mentioned above, only administration of Ang I, Ang II, and Ang-(1-12) caused a substantial increase in SBP in WT mice (Figure 3). Since these angiotensin peptides are all cleavable by APA (Figure 2), we then examined whether the administration of murine rAPA to WT mice modified their pressor effect.

**Figure 4:**
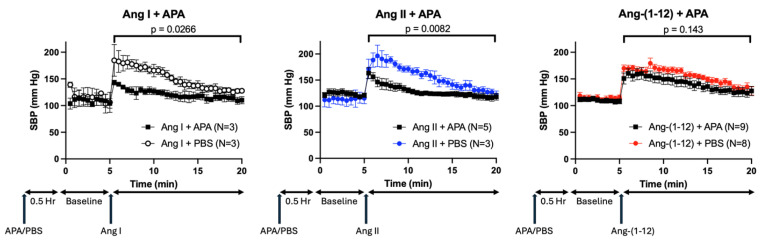
Systolic blood pressure in WT mice after injection of Ang I, Ang II, and Ang-(1-12) pre-administered with recombinant (r)APA or vehicle (PBS). Systolic blood pressure (SBP) measured before and after injection of 0.2 μg/g BW Ang I, Ang II, and Ang-(1-12) (arrow) in male C57BL/6J mice which were pre-administrated with recombinant (r)APA (1 μg/g BW) or vehicle (PBS) 30 min before SBP measurement. (**Left**): Recombinant APA significantly enhances the rate of recovery from the SBP elevation induced by Ang I (*p* = 0.0266) compared to PBS (N = 3 vs. N = 3). (**Middle**): Recombinant APA significantly enhances the rate of recovery from the SBP elevation induced by Ang II (*p* = 0.0082) compared to PBS (N = 5 vs. N = 3). (**Right**): Recombinant APA does not significantly enhance the rate of recovery from the SBP elevation induced by Ang-(1-12) (*p* = 0.1403) compared to PBS (N = 9 vs. N = 8).

Immediately after each of the Ang peptides’ injection, there was a peak in SBP followed by a slow decline. The curves of SBP decline after Ang-peptide injection were compared between murine rAPA- and PBS-preinjected (vehicle) mice using two-way ANOVA. Recombinant APA accelerated the return of the SBP to pre-infusion levels elicited by Ang I (Figure 4 in the left panel) and Ang II (Figure 4 in the middle panel) (*p* = 0.0266 and *p* = 0.0082, respectively). In contrast, rAPA did not result in a faster recovery from Ang-(1-12)-induced SBP increase as compared to PBS-preinjected mice (Figure 4 in the right panel).

Ang I is believed to have no biological action by itself but rather through the formation of Ang II, and there is uncertainty whether Ang-(1-12) can act directly or indirectly via Ang II. Therefore, further studies in mice aimed to compare only the effects of Ang II- and Ang-(1-12)-induced SBP increases.

### 2.4. Effect of Acute Ang II and Ang-(1-12) Infusion on Systolic Blood Pressure in a Model of Global APA Deficiency

**Figure 5:**
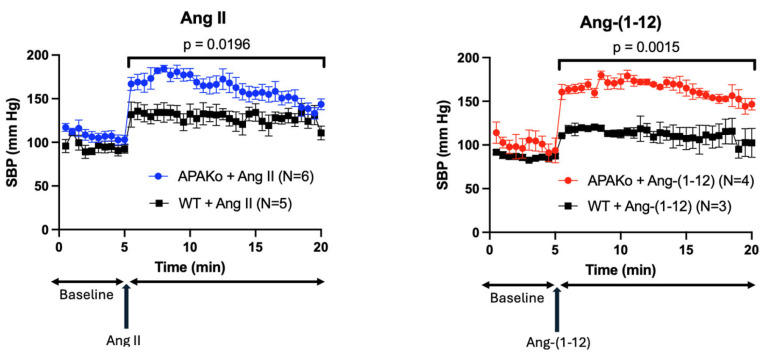
Systolic blood pressure in WT and APA Ko mice before and after injection of Ang II and Ang-(1-12). Systolic blood pressure (SBP) in WT (squares) and APAKO mice (circles) before (baseline) and after injection of 0.2 μg/g BW (arrow) of Ang II (**left** panel) or Ang-(1-12) (**right** panel). (**Left**): Ang II causes a significantly bigger increase in SBP in APAKO as compared to WT mice (*p* = 0.0196) (N = 6 vs. N = 5). (**Right**): Ang-(1-12) causes a significantly bigger increase in SBP in APAKO as compared to WT mice (*p* = 0.0015) (N = 4 vs. N = 3).

Male WT mice and male mice with global APA deficiency were injected i.p. with the same dose (0.2 μg/g BW) of Ang II or Ang-(1-12). Compared to WT mice, Ang II caused an exaggerated increase in SBP in APAKO mice (Figure 5). Similarly, when the same Ang-(1-12) dose was injected, the SBP increase was significantly larger and normalized more slowly in APAKO mice than in WT counterparts (Figure 5). This shows the critical importance of APA in controlling blood pressure when Ang II and Ang-(1-12) are present in large amounts.

### 2.5. Effect of ACE Inhibitor and AT1 Receptor Blocker on Ang-(1-12) Infusion

**Figure 6:**
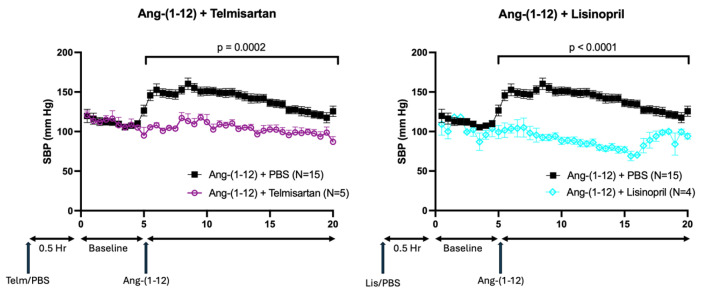
Effect of Telmisartan (Telm) and Lisinopril (Lis) on injection of Ang-(1-12) in WT mice. The effect of the AT1 receptor blocker, Telmisartan (1 μg/g BW, **left** panel), and ACE inhibitor Lisinopril (1 μg/g BW, **right** panel) on systolic blood pressure (SBP) responses caused by the injection of Ang-(1-12) (0.2 μg/g BW, arrow). Telmisartan and Lisinopril were injected 30 min before SBP measurements. (**Left**): The SBP increase caused by Ang-(1-12) injection (arrow) is completely blocked by Telmisartan as compared to the PBS pre-injected control group (*p* = 0.0002) (N = 15 vs. N = 5). (**Right**): The SBP increase caused by Ang-(1-12) injection (arrow) is completely blocked by Lisinopril as compared to the PBS pre-injected control group (*p* < 0.0001) (N = 15 vs. N = 4).

ACE inhibitors and AT1 receptor blockers can considerably attenuate Ang II formation or its action, respectively. We examined the effect of Lisinopril (an ACE inhibitor) and Telmisartan (AT1 receptor blocker) on blood pressure responses elicited by Ang-(1-12) to prevent Ang-(1-12)-induced Ang II formation or AT1 receptor stimulation. WT mice were pre-injected with Lisinopril (1 μg/g BW) or Telmisartan (1 μg/g BW), 30 min before i.p. injection of Ang-(1-12). Both Lisinopril and Telmisartan completely abolished the SBP increase caused by Ang-(1-12) (*p* < 0.01) (Figure 6).

### 2.6. Effect of rACE2 on Ang-(1-12) Infusion

**Figure 7:**
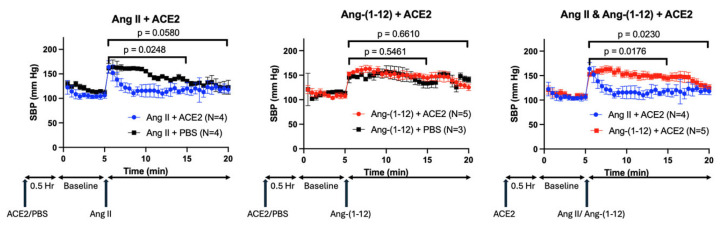
Effect of recombinant mouse ACE2 (740) on systolic blood pressure increases induced by Ang II and Ang-(1-12) injection. The effect of recombinant mouse ACE2 (1 μg/g BW) on systolic blood pressure (SBP) responses caused by the injection of Ang II (left panel), Ang-(1-12) (middle panel) (both 0.2 μg/g BW, arrow), and Ang II and Ang-(1-12) superimposed over each other. (**Left**): Compared to a PBS pre-injected control group, rACE2 reduced the SBP response to Ang II over ten minutes after injection by *p* = 0.0248 (rACE2) by 2-way ANOVA. When comparing over fifteen minutes, the injection of rACE2 reduced the SBP response by *p* = 0.058 (N = 4 vs. N = 4). (**Middle**): As compared to a PBS pre-injected control group, rACE2 did not reduce the SBP response to Ang-(1-12) either over ten minutes after injection (*p* = 0.5461) or over fifteen minutes after injection (*p* = 0.6610) (N = 5 vs. N = 3). (**Right**): Direct comparison of the effect of rACE2 on reducing the SBP response between the Ang II-induced (blue) and Ang-(1-12) (red)-induced SBP increase which was significantly different both over ten minutes from the injection (*p* = 0.0176) and over fifteen minutes from the injection of the peptides (*p* = 0.0230) (N = 4 vs. N = 5).

We reasoned that, if the Ang-(1-12) effect on blood pressure is indeed mediated by Ang II formed from Ang-(1-12), then ACE2, which degrades Ang II to Ang-(1-7), would abolish the Ang-(1-12) pressor action. WT mice were pre-treated with rACE2 30 min before injecting either Ang II or Ang-(1-12) (Figure 7). The hypertensive effect of Ang II was quickly abolished by the administration of rACE2 (*p* = 0.02), whereas the hypertensive effect of Ang-(1-12) remained largely unaffected by rACE2 (*p* = 0.66).

## 3. Discussion

This study shows that aminopeptidase A can consistently cleave the N-terminal aspartate residue from all angiotensin peptides we tested. This was demonstrated using a novel in vitro assay that we developed to quantitatively measure free aspartate formation as a readout of the enzymatic cleavage of different Ang peptides by recombinant (r)APA. The removal of aspartate for a broader range of Ang peptides with N-terminal aspartate has not been tested systematically using recombinant APA. By employing our new in vitro method, we were able to unequivocally show that not only Ang I (1-10) and Ang II (1-8), but also several other Ang peptides bearing N-terminal aspartate, such as Ang-(1-7), Ang-(1-9), and Ang-(1-12), are cleaved by rAPA with a comparable efficiency. This finding, moreover, was demonstrated for both mouse and human rAPA (Figure 2 and Figure S1). In contrast to murine and human rAPA, substantial species differences in cleavage efficiency have been reported for rACE2. Murine rACE2 cleaved Ang I (1-10) very inefficiently as compared to human rACE2 [7,20]. Also, unlike rAPA, rACE2 exhibited significant differences in the cleavage efficiency of different angiotensin substrate peptides [7]. Whereas in this study no appreciable differences could be observed between the cleavage efficiency of Ang II and Ang I by rAPA, we confirmed that rACE2 cleaves Ang II to Ang-(1-7) with a much higher catalytic efficiency than the comparative hydrolysis of Ang I to Ang-(1-9) [7] consistent with earlier findings [21,22]. The biological importance of the N-terminal processing of Ang peptides became evident from the finding that conversion rates of kidney Ang I to Ang II to biologically inactive products in rats were found to be about 60% dependent on aminopeptidases [23].

**Figure 8:**
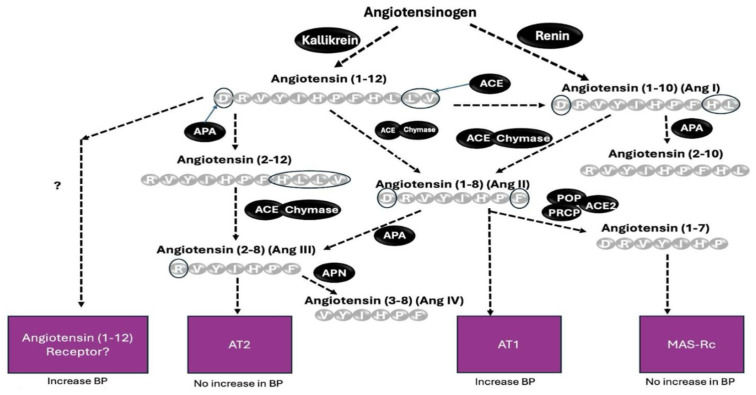
Metabolism of Angiotensin (1-12) by APA and other enzymes may support the downstream formation of prohypertensive and non-hypertensive peptides. This process can be renin-independent. A hypothetical Ang-(1-12) receptor is indicated to reflect the possibility of a direct signaling mechanism, independent of known metabolites such as Ang II.

Among angiotensin peptides, the cleavage of N-terminal aspartate by APA has been previously shown for the octapeptide Ang II (1-8), resulting in the formation of the heptapeptide Ang-(2-8) [24,25,26] and also Ang 1 (1-10) to form Ang (2-10) [14]. Aminopeptidase A has also been implicated in the cleavage of Ang I (1-10) to Ang (2-10), as inferred from in vitro processing of Ang I (1-10) by cultured podocytes in the presence and absence of APA inhibitor [12]. In this study, we provide new in vitro and in vivo data to expand the understanding of the contribution of aminopeptidase A (APA) to the systemic pressor effect of various Ang peptides (Figure 8). This adds information not provided by previous studies [27]. To fill this gap, we examined the pressor effects of systemic infusions of Ang peptides, identified as rAPA substrates, in our in vitro assay. We found that systemic administration of Ang-(1-9) and Ang-(1-7) to wild type mice did not cause any SBP increases (Figure 3). This is in agreement with previous studies revealing no vasoconstrictor actions of either Ang-(1-7) [6,28] or Ang-(1-9) [29]. As expected, the infusion of Ang I, Ang II, and Ang-(1-12) using the same concentration as the other two peptides had a potent acute prohypertensive effect. It is also known that the potent vasopressor peptide Ang II, when converted by aminopeptidases such as APA to Ang III (2-8) [27], loses much of its pressor potency during systemic infusion [30]. It should be noted, however, that during intracerebroventricular infusion [31] both peptides may be equipotent in raising blood pressure [31]. Our goal was to examine whether APA could influence acute angiotensin-dependent hypertension by controlling the degradation of not only Ang II but also other RAS peptides, including Ang-(1-12). We found that rAPA substantially blunted the acute blood pressure increases elicited by Ang I and Ang II. In addition, a faster recovery to baseline blood pressure levels was achieved for Ang I and Ang II. For Ang (1-12), however, only a weak and insignificant effect of rAPA was observed, as the blood pressure decline velocity did not reach statistical significance. Moreover, the BP peak after Ang-(1-12) injection was similar with and without rAPA (Figure 7).

We reasoned that global APA deficiency could exaggerate the acute hypertensive responses elicited by various Ang peptides. In global APA-deficient mice, we demonstrated that the acute hypertensive effect of Ang-(1-12), like that of Ang II, was greatly amplified compared to WT (Figure 5). However, rAPA infusion in WT mice, to increase the circulating APA levels, was able to reduce Ang II-induced BP increase but failed to reduce the acute BP increase caused by Ang-(1-12) infusion (Figure 4). This contrasts with the effect of global APA deficiency on Ang-(1-12)-induced SBP spikes.

Angiotensin (1-12) may serve as a substrate for the downstream formation of several peptides, as shown in Figure 8. This peptide gained attention when it was found that it is formed from angiotensinogen in a renin-independent manner [32]. It was later recognized that Ang-(1-12) can be converted to Ang II [33,34,35] by the action of ACE and chymase [35]. However, it remains unclear whether the biologic effect of Ang-(1-12) is solely associated with Ang II formation [36] and the binding of Ang II to the AT1 receptor [36] or whether Ang-(1-12) itself is capable of directly binding to the AT1-receptor [37]. In support of the latter possibility, Chan et al. [37] presented evidence that Ang-(1-12) can act directly on the AT1-receptor in cells transfected with AT1R, though less robustly than Ang II. It is also plausible that Ang-(1-12) could act via its own receptor for which currently there is no evidence. In this study, we found that the Ang-(1-12) pressor effect is prevented by RAS blockade, consistent with action dependent on Ang II formation and activation of the AT-1 receptor as reported previously [35]. Of interest, however, the administration of rACE2, which is known to blunt the hypertensive effect of Ang II [6,7,9] did not decrease significantly the hypertensive effect of Ang-(1-12) (Figure 7). This finding suggests a direct hypertensive action of Ang-(1-12), which, to a certain extent, is independent of its conversion into Ang II. In addition, it is possible that APA-mediated degradation of Ang-(1-12) could lead to the formation of peptide products exerting hypertensive action. Indeed, ex vivo studies using human blood plasma samples suggested the existence of an aminopeptidase-mediated pathway that bypasses the known classic Ang I (1-10) to Ang II (1-8) conversion pathway by ACE [24]. In this context, it is interesting to note that the infusion of Ang-(2-10) was found to have a modest pressor effect [38]. Ang-(2-10) is a direct product of APA action on Ang I (1-10) but could also possibly be a product of the joint action of aminopeptidases and ACE on Ang-(1-12) [24].

The mechanism responsible for this partial independence from Ang II remains unresolved. Although the prevailing view attributes the cardiovascular effects of Ang-(1-12) to its enzymatic conversion into Ang II and subsequent stimulation of AT1 receptors, our data—along with other findings [37]—suggest that Ang-(1-12) might exert direct actions. These could involve low-affinity binding to AT1 receptors at elevated concentrations [37] or result from alternative pathways involving receptor types that remain to be identified, or from bioactive fragments produced via APA-dependent cleavage. Clarifying whether Ang-(1-12) interacts with AT1 receptors directly, acts via a novel receptor, or generates active metabolites with pressor capacity will require further investigation [39,40]. The notion of an Ang II-independent mechanism gains support from studies reporting that Ang-(1-12)-induced cardiovascular changes are only partially attenuated by ACE inhibition or the AT1 receptor blockade [40,41,42,43,44]. Consistent with these observations, our own experiments revealed that the pressor response to Ang-(1-12) persisted despite the presence of APA or ACE2—enzymes known to efficiently degrade Ang II—implying a distinct pharmacological profile. Although dose–response experiments were no part of this study, the data support the hypothesis that Ang-(1-12) may influence blood pressure regulation through a unique, potentially dose-dependent mechanism that extends beyond the traditional AT1R axis. Further work is needed to examine the metabolism of this peptide in vivo and its role in the RAS. We acknowledge that, while Ang-(1-12) is measurable in plasma in significant amounts by radioimmunoassay [33,45], one recent report found it difficult to measure it in detectable amounts using liquid chromatography-tandem mass spectrometry [46].

In conclusion, our findings underscore the complexity of the RAS network and highlight the necessity for a more comprehensive understanding of its various components to develop more efficacious therapeutic strategies for hypertension. Current mainstay therapies to control blood pressure targeting RAS focus on blocking the formation of Ang II or its AT1 receptor. Our results show the importance of aminopeptidase A as a potential approach to reduce the pressor effects of Ang II and a role as a treatment for forms of Ang II-dependent hypertension. Angiotensin (1-12) metabolism initiated by APA and other enzymes can provide a source of activation of the RAS cascade, which is renin-independent. We now show that the pressor action of Ang-(1-12) is partly independent of the formation of Ang II since it was not prevented by enzymes that rapidly metabolize it, such as APA and ACE2.

## 4. Materials and Methods

### 4.1. In Vitro Assessment of Cleavage of N-Terminal Aspartate from Various Angiotensin Peptides by Recombinant APA

To identify Ang peptides cleaved by rAPA, we developed a novel in vitro assay that measures quantitatively free aspartate formation from the Ang peptides incubated with recombinant APA. In this assay, the amount of cleaved aspartate from the N-terminus of those Ang peptides by rAPA is captured using Sigma-Aldrich kit, Saint Louis, MO, USA (Cat # MAK095-1KT) and is proportional to the recorded fluorescence expressed in relative fluorescence units (RFU). A 293-cell line (CRL-1573) was used to produce soluble mouse recombinant APA for stable transfection and clone isolation. Serum-free medium collected from the isolated clone over-expressing murine rAPA was buffer-exchanged against 20 mM Tris-HCl, pH 8.0, and then subjected to anion exchange chromatography using Q-sepharose (Cytiva) on Cytiva FPLC chromatography system.

To determine the dynamic performance of the assay, fluorescence was measured for serially diluted rAPA (ranging from 200 ng to 1.56 ng/well) containing the same amount of Ang II over a period of 60 min. There was a complete dose separation for every condition proving a relative dependence on the amount of rAPA as an Asp-cleaving enzyme (Figure S3). Then, by incubating a fixed amount of rAPA with differing amounts of Ang II, we derived a dose–response curve for the substrate (presented in Figure S4.).

Angiotensin peptides (Ang-(1-7), Ang-(1-9), Ang-(1-12), Ang I, and Ang II (Bachem, Inc., Bubendorf, Switzerland, catalog numbers 4006081, 4033395, 4061269, 4002346, and 4006473)) for the in vitro reaction were used at a concentration of 10 nmol, in the presence of the purified murine recombinant (r)APA (100 fmol) or human rAPA (R&D Systems, Minneapolis, MN, USA, 100 fmol) for 1 h at room temperature in a total of 100 µL of volume in Tris-buffered saline (TBS) at a pH of 7.4. To assure the validity of the assay, several controls were included. Recombinant APA (mouse and human) was incubated without any peptide to account for background fluorescence. Other background controls included wells where each tested peptide was incubated without rAPA in the reaction mix. In addition, two peptides lacking aspartate at the N-terminus were incubated with rAPA as negative controls: Angiotensin A (Bachem, Inc.) which is an octapeptide homologue of Ang II [18] and Alamandine (Bachem, Inc.) a heptapeptide homologue of Ang-(1-7) [19] (in both peptides, Alanine is in position 1 instead of aspartate). In those two peptides, no aspartate cleavage by APA was expected. Moreover, recombinant ACE2 (rACE2) (R&D Systems, 100 fmol), a mono-carboxypeptidase enzyme that does not cleave N-terminal aspartate but cleaves C-terminal basic and hydrophobic amino acids [22], was used as another negative control to show that, when incubated with Ang peptides bearing N-terminal aspartate, no fluorescent signal can be produced. Finally, mouse rAPA was incubated with Ang peptides in the presence and absence of amastatin (10^−5^ M end concentration) to ascertain whether the fluorescence reaction is inhibitable by an APA inhibitor.

### 4.2. In Vivo Studies

All animal studies performed with mice were approved by the Northwestern University Animal Care and Use Committee (protocol number IS00004795). Mice were chosen for these in vivo studies because our blood pressure measurement protocols have been previously validated in this species [6,7,8,9].

### 4.3. Blood Pressure Measurement

To measure systolic blood pressure (SBP) noninvasively in anesthetized mice, a previously described method [6,7] was utilized. Systolic blood pressure was measured by a tail cuff using a computerized system (CODA System, Kent Scientific, Torrington, CT, USA). This volume-pressure recording system has been validated for SBP measurements and demonstrated a strong correlation with telemetry and direct arterial blood pressure measurements [47]. Because in male mice Ang II induces more consistent SBP increases than in female mice [48,49], SBP responses to various Ang peptides were studied only in male mice. To determine acute effect of different Ang peptides on the SBP, male *C57BL/6J* mice were first anesthetized with an IP ketamine injection (133 mg/kg of body weight (BW)) and placed on a heating platform for 10 min. The SBP was then measured every 30 s for 20 min: after 5 min of baseline SBP recording, a bolus of 5 different Ang peptides (Ang-(1-7), -(1-9), -(1-12), I or II at 0.2 μg/g BW), each bearing N-terminal aspartate, was injected intraperitoneally to 5 different groups of mice. Delta SBP was calculated by subtracting the means of the SBP values measured immediately 5 min before and 5 min after angiotensin peptides bolus injection.

To investigate the effect of APA deficiency on acute Ang-induced SBP spikes, total body male APA knockout (ko) mice and their male WT littermates on the *BALB/c* background were used for the same experimental setup. The generation of this APAko was previously reported [17], and breeding pairs were kindly provided to us, by Dr. J-C. Velez (the Medical University of South Carolina). This mouse model has been characterized previously [11,13].

To study how various RAS enzymes or enzyme inhibitors (such as rAPA, rACE2, Telmisartan, and Lisinopril) alter the acute effect of different angiotensin peptides on systolic blood pressure (SBP), male *C57BL/6J* mice were anesthetized with an IP ketamine injection (133 mg/kg of body weight). Thirty minutes before anesthesia, mice were pretreated with an IP injection of either sterile PBS (0.4 mL) as a control or rAPA (1 μg/g BW), Telmisartan (1 μg/g BW), Lisinopril (1 μg/g BW), or rACE2 (1 μg/g BW). Immediately after inducing anesthesia, mice were placed on a heating platform for 10 min. SBP was then monitored for 20 min. After 5 min of baseline SBP recording, an IP bolus of Ang peptides (Ang-(1-12), Ang I, or Ang II (0.2 μg/g BW) was given, and the SBP was monitored for the remaining 15 min.

### 4.4. Statistical Analysis

We used Windows Excel 2016 (Microsoft, Redmond, WA), and GraphPad Prism 10 (GraphPad Software, San Diego, CA, USA) for data analysis and generation of graphs. Normally distributed data were analyzed by *t*-test (2 groups) or ANOVA (more than 2 groups) followed by Dunnett’s multiple comparisons test. Changes between groups over time were analyzed using 2-way ANOVA followed by Tukey’s multiple groups comparisons. A *p*-value of less than 0.05 was considered significant.

## Data Availability

Source data for this study are available upon request.

## Supplementary Materials

The following supporting information can be downloaded at https://www.mdpi.com/article/10.3390/ijms26146990/s1.
