# Supplementary material for: Aminopeptidase A Effect on Angiotensin Peptides and Their Blood Pressure Action
Source: Int J Mol Sci. 2025 Jul 21;26(14):6990. doi: 10.3390/ijms26146990 (PMC12296148; doi:10.3390/ijms26146990)
Supplement: Supplementary file 1 [file ijms-26-06990-s001.zip › ijms-3743123-supplementary.pdf]

Supplemental Information

Title: Aminopeptidase A effect on Angiotensin Peptides and their blood pressure action.

Peter Forster[1,2], Jan Wysocki[1], Yasemin Abedini[1], Tilman Müller[1], Minghao Ye[1], Carlos M. Ferrario[3], Daniel Batlle[1]

[1]Division of Nephrology and Hypertension, Department of Medicine, Northwestern University Feinberg School of Medicine, Chicago, IL, USA. [2]Charité-Universitätsmedizin Berlin, Germany. [3]Wake Forest University School of Medicine, USA.

Corresponding author
Daniel Batlle
Division of Nephrology and Hypertension
Northwestern University Feinberg School of Medicine
710 N Fairbanks Court
Chicago, IL, 60611
Phone: (312) 908-8342
d-battle@northwestern.edu

Supplemental Figures

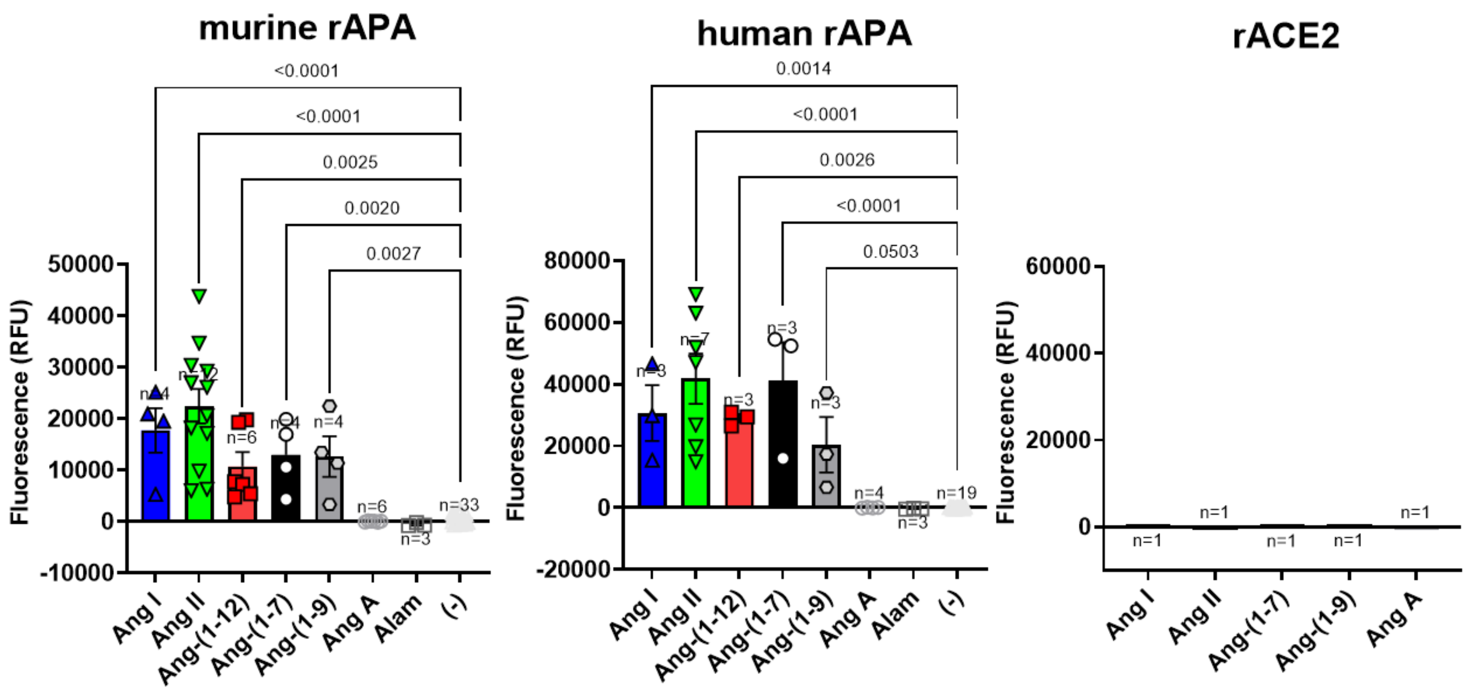

Supplemental Figure S1. Cleavage of N-terminal aspartate from various angiotensin peptides by murine recombinant aminopeptidase A (rAPA) (left panel), human rAPA (middle panel) and recombinant angiotensin-converting enzyme 2 (rACE2) (right panel) measured by fluorescence formation using the *in vitro* assay described in the Methods. Each bar represents the mean ± standard error with an n of experiments indicated above or below the bar for each of the peptide tested. Ang A – angiotensin A; Alam – Alamandine, (-) – rAPA without peptide substrate. The p values were calculated using One-Way Anova followed by Dunnett's multiple comparison test.

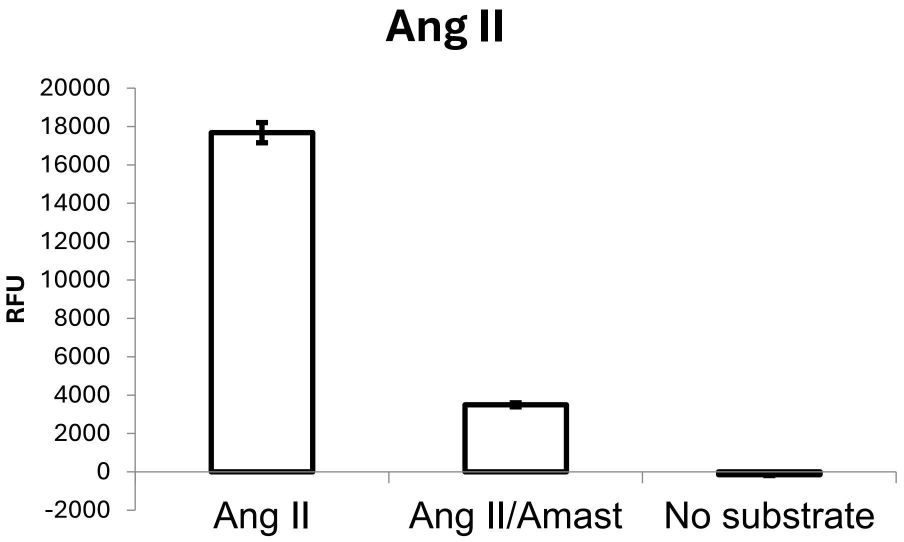

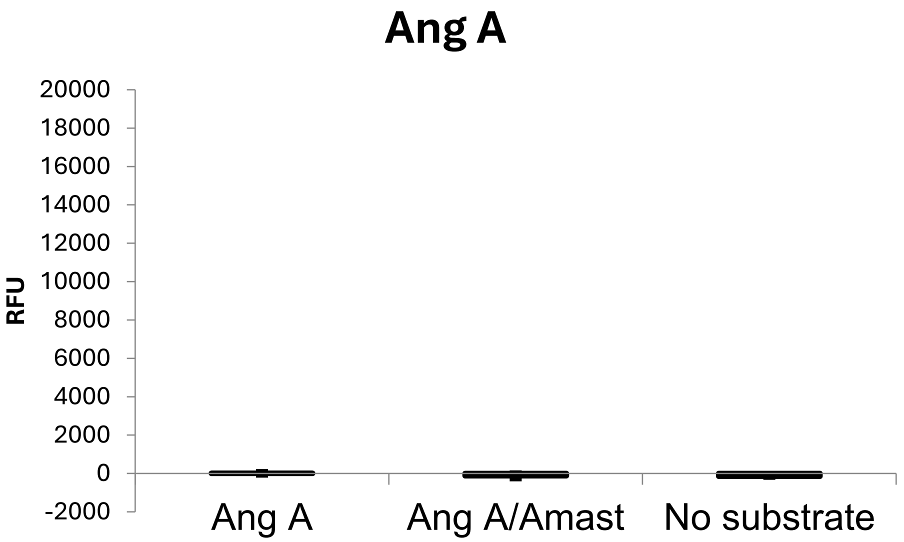

Supplemental Figure S2. Murine recombinant aminopeptidase A (rAPA) was incubated with Ang II (upper panel) and with Angiotensin A (lower panel) in the absence and presence of amastatin, an APA inhibitor (10^{-5}M end concentration) and the cleavage of N-terminal aspartate from the two angiotensin peptides was measured by fluorescence formation using the *in vitro* assay described in the Methods. Murine rAPA without the respective angiotensin peptide was used as a negative control that could account for the background formation of free aspartate from the rAPA without the presence of the substrate peptide. Each bar represent a mean of two experiments.

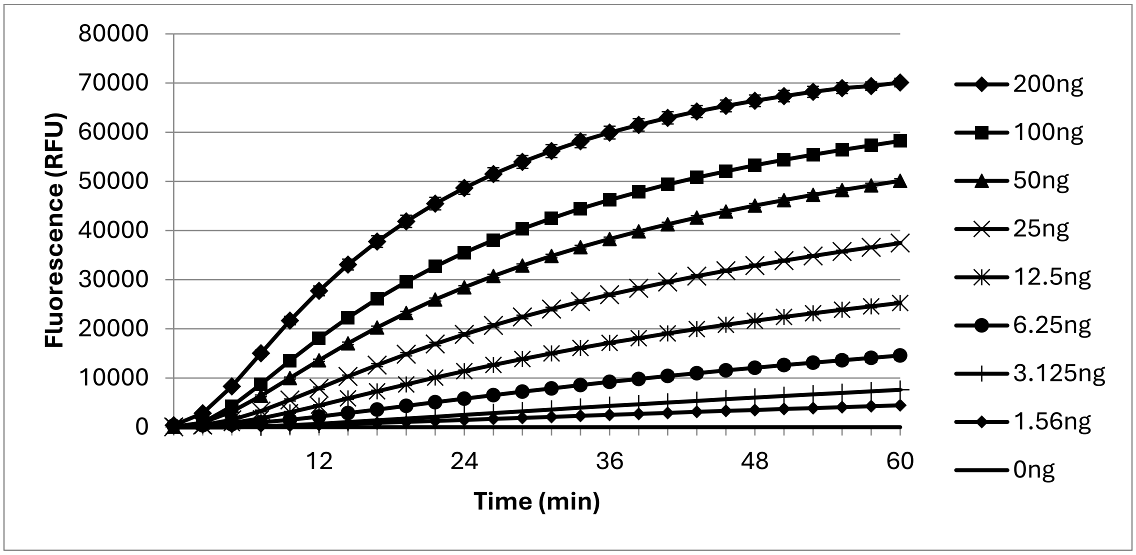

Supplemental Figure S3. Recombinant aminopeptidase A (rAPA) was serially diluted and incubated with the same amount of Ang II (10 nmol) and fluorescence recorded over 60 minutes at room temperature. Free aspartate formation was measured through the formation of a fluorometric product at 535 excitation and 585 emission wave lengths using reagents from the Sigma-Aldrich kit (Cat # MAK095-1KT).

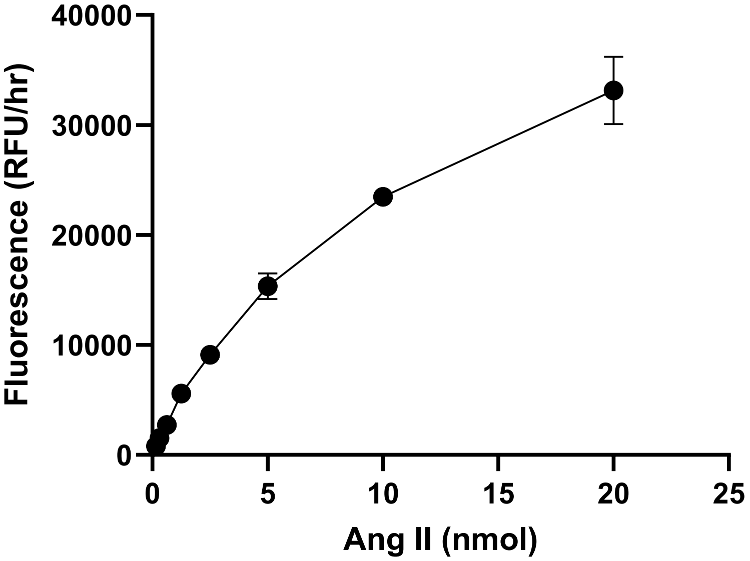

Supplemental Figure S4. Different amounts of Ang II (up to 20 nmol) were incubated with recombinant aminopeptidase A (at a single end-concentration of 100 ng/well). Free aspartate formation was measured through the formation of a fluorometric product at 535 excitation and 585 emission wave lengths using reagents from the Sigma-Aldrich kit (Cat # MAK095-1KT).
